# Preoperative MRI Predictors for Post-Prostatectomy Urinary Incontinence

**DOI:** 10.3390/cancers17183004

**Published:** 2025-09-15

**Authors:** Franco Alchiede Simonato, Guglielmo Mantica, Martina Beverini, Francesca Ambrosini, Francesco Chierigo, Veronica Giasotto, Nicola Pavan, Alchiede Simonato, Carlo Terrone

**Affiliations:** 1 Urology Clinic, Department of Precision Medicine in Medical, Surgical and Critical Care, University of Palermo, 90127 Palermo, Italy; francoalchiede@gmail.com (F.A.S.); nicpavan@gmail.com (N.P.); alchiede@gmail.com (A.S.); 2 Department of Surgical and Diagnostic Integrated Sciences (DISC), University of Genoa, 16126 Genova, Italy; carlo.terrone@unige.it; 3 IRCCS Ospedale Policlinico San Martino, 16132 Genova, Italyveronicagiasotto@gmail.com (V.G.); 4 Department of Urology, UMC+, 6229 ER Maastricht, The Netherlands; f.ambrosini1@gmail.com; 5 Department of Urology, ASST Santi Paolo E Carlo, University of Milan, 20133 Milan, Italy

**Keywords:** post-prostatectomy incontinence, MRI predictors, prostatic apex shape, urethral length, median lobe

## Abstract

**Background**: We investigated the predictive role of prostatic morphology on preoperative multiparametric magnetic resonance imaging for post-prostatectomy urinary incontinence. **Methods**: Patients who underwent robot-assisted radical prostatectomy between February 2018 and October 2021 and who were not previously incontinent, did not undergo radiotherapy, hormone therapy, or transurethral resection of the prostate, and who had a follow-up longer than 12 months were selected. For each patient, a radiology physician evaluated the preoperative magnetic resonance imaging, measuring prostatic and membranous urethral length, classifying prostatic apex according to the Lee Type, and estimating the presence of the median lobe and its intravesical protrusion. Multivariate logistic regression models evaluated the influence of anatomic features measured in magnetic resonance imaging on urinary continence recovery, defined as daily pad usage less than or equal to one, considering age, body mass index, prostate volume, International Prostatic Symptoms Score, the usage of a nerve sparing technique, and the International Society of Urological Pathology classification. **Results**: A total of 95 patients who underwent robot-assisted radical prostatectomy were enrolled. Median age, median body mass index, and median PSA density were respectively 66 years (62, 70), 26.12 kg/m^2^ (23.88, 28.09), and 0.16 ng/mL/cc (0.10, 0.26). Patients with urinary continence ranged from 32 (33.7%) at baseline to 93 (97.8%) after one year from surgery. At preoperative magnetic resonance, Lee Type was almost equally distributed, but Type C was less represented (18 patients, 18.9%) and Type D was more frequent (31 patients, 32.2%). Median prostatic urethral length, median membranous urethral length, and median intravesical prostatic protrusion were respectively 36 mm (31, 42), 15 mm (13, 16), and 0 mm (0, 0). Multivariate logistic regression models showed no statistical significance, except for Lee Type C and A comparison at vesical catheter removal after surgery (OR 0.17; 95% CI 0.04–0.71; *p*-value 0.01). **Conclusions**: The results of this study showed that patients who had Lee Type C might have higher probability of early urinary continence recovery, but no further statistically significant correlations were found.

## Simple Summary

This study investigated whether anatomical features seen on preoperative multiparametric MRI could predict urinary continence recovery after robot-assisted radical prostatectomy. The analysis included 95 patients who were continent before surgery and had at least 12 months of follow-up. Radiological evaluations measured urethral lengths, assessed prostatic apex shape (Lee classification), and the presence of median lobe protrusion. Overall, urinary continence improved from 33.7% at baseline to 97.8% one year after surgery. Most MRI-based features did not significantly predict continence recovery, except for the Lee Type C apex, which was associated with better early recovery compared to Type A (OR 0.17; *p* = 0.01). In conclusion, while most anatomical MRI features were not predictive, the shape of the prostatic apex (Lee Type C) may be linked to earlier continence restoration.

## 1. Introduction

In recent years, the diagnostic and therapeutic approach to prostate cancer (PCa) has undergone significant advancements [1,2]. The traditional reliance on systematic biopsies and Prostate Specific Antigen (PSA) alone has been progressively replaced by more precise and individualized strategies [3,4]. The widespread use of multiparametric magnetic resonance imaging (mpMRI), often combined with targeted fusion-guided biopsies, has significantly improved the detection and risk stratification of clinically significant PCa. In parallel, the integration of novel imaging modalities, such as PSMA PET/CT, has enhanced staging accuracy, particularly for high-risk and recurrent disease [5]. Furthermore, the increasing availability of nomograms, risk calculators, and mobile health applications has facilitated shared decision-making and tailored treatment planning, empowering both patients and clinicians in choosing the most appropriate therapeutic pathway [6,7,8,9,10,11,12].

Currently, radical prostatectomy (RP) remains the gold standard surgical treatment for localized PCa disease. Over the years, RP has evolved significantly in terms of surgical technique, perioperative outcomes, and oncological efficacy, especially with the adoption of minimally invasive and robotic-assisted approaches [13,14,15]. This procedure, performed either via open, laparoscopic, or robot-assisted approaches, aims to achieve complete oncological control while minimizing functional side effects. One of the most common complications of RP is urinary incontinence (UI), which reduces quality of life (QoL) of patients treated [16,17,18,19]. In particular, UI can persist for weeks or months after surgery and may require the use of pads, behavioral therapy, or even surgical interventions, significantly impacting daily activities and psychological well-being [20,21].

Due to the high frequency of post-RP UI and the reduced QoL, possible predictors were recently studied in order to better stratify patients for the most appropriate treatment. In this context, identifying preoperative factors that may influence functional outcomes has become a crucial goal in urological research and clinical practice. Better risk stratification may help clinicians select patients who could benefit from specific counseling, early rehabilitation, or nerve-sparing techniques. Possible predictors of urinary continence (UC) recovery, evaluated from preoperative magnetic resonance imaging (MRI), might be prostatic apex shape according to Lee type (LT), median lobe (ML) presence and its intravesical protrusion (IPP), prostatic urethral length (PUL), or membranous urethral length (MUL) [17,18,22,23,24,25,26]. These parameters are measurable with high reproducibility and may provide objective data to guide surgical planning and anticipate postoperative functional results.

Other possible predictors might be age, body mass index (BMI), International Prostate Symptoms Score (IPSS), Gleason Score (GS), disease stage, and use of a nerve sparing technique (NST) during RP [23,24,27]. These variables, which are routinely collected in the preoperative work-up, may affect both oncological and functional outcomes, and their potential role in continence recovery is increasingly recognized in the literature. For instance, younger patients or those undergoing bilateral nerve sparing might have better chances of regaining continence earlier.

We aimed to find predictors of post-RP UC recovery and how much they could affect it, in order to better identify high UI risk patients who might benefit from specific treatments. The ultimate goal is to move toward a more personalized approach to prostate cancer surgery, where the choice of technique and the timing of rehabilitation can be tailored according to individual risk profiles. This could improve patient satisfaction, reduce anxiety, and promote a faster return to normal life.

## 2. Materials and Methods

A monocentric retrospective study was designed and the primary outcome was defined as the UC recovery at 0, 1, 3, and 6 months after robot-assisted radical prostatectomy (RARP) in PCa patients.

PCa patients who underwent RP between February 2018 and October 2021 were included. Patients who were not treated with RARP, with no available MRI, with follow-up shorter than 12 months, who underwent transurethral resection of the prostate (TURP) or other PCa treatment (such as radiotherapy, hormone therapy, cryotherapy, and/or focused high intensity ultrasound), or with a MRI where parameters could not be accurately measured were excluded.

UC recovery was defined as a daily pad usage less than or equal to one.

**Figure 1:**
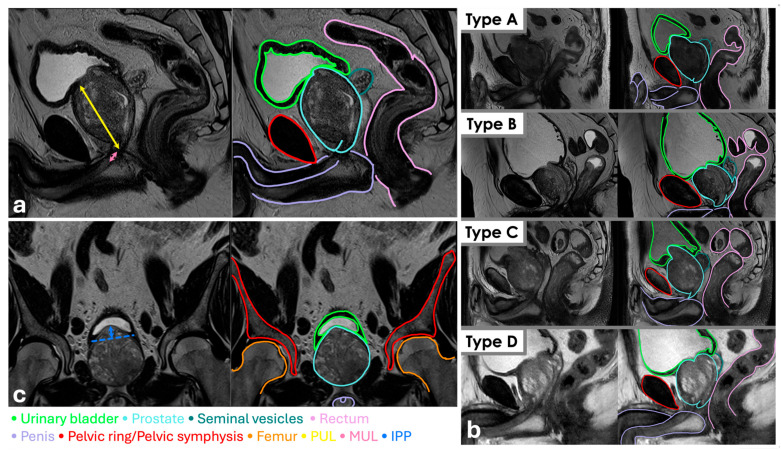
Multiparametric magnetic resonance imaging (MRI) of the male pelvis with anatomical annotation and morphological Lee Types classification. (**a**) Sagittal views; (**b**) Examples of different sagittal morphological variants of the urethro–prostatic junction and pelvic relationships (Types A–D); (**c**) Coronal views.

For every patient, at the diagnosis, age, weight, height, body mass index (calculated as quotient between weight and height squared), PSA, PSA Density (PSAD), GS at biopsy, ISUP, clinic stage, IPSS, and QoL were collected. An expert radiologist (V.G.), blinded to postoperative outcomes, evaluated every MRI, estimating prostatic volume (PV), PUL, MUL, prostatic apex shape, ML presence, and IPP. PV was calculated as the product among π/6 and the three maximum diameters measured at T2 weighted images in sagittal and axial sections. PUL was measured from prostatic base to apex at T2 weighted images in sagittal section (Figure 1a) and MUL from prostatic apex to urethral bulb at T2 weighted images in sagittal section (Figure 1a). Apex shape was classified according to LT at T2 weighted images in sagittal section (Figure 1b) and IPP was measured from intravesical protrusion apex and vesical base at T2 weighted images in coronal section (Figure 1c).

During RARP, the execution of a NST or a bladder neck sparing technique was collected. During follow-up, every patient was encouraged to do Kegel’s exercises in order to strengthen their pelvic floor and was questioned about their daily pad usage.

Descriptive tables show clinical and radiological data with median and interquartile range (IQR), for continuous variable, or absolute number with percentage of the total, for categorical variables. Multivariate logistic regressions were performed to test predictors for UC recovery at vesical catheter (VC) removal, 1, 3, and 6 months from RARP. Covariates included age, BMI, PV, IPSS (all continuously coded), NST (yes vs. no), ISUP (1 vs. 2 vs. 3 vs. 4), PUL, MUL, ML, IPP (continuously coded), and LT (A vs. B vs. C vs. D) as independent variables. Statistical results were presented as odds ratios (ORs) with 95% confidence interval (95% CI) and *p*-value considered statistically significant if less than or equal to 0.05. Statistical analyses were conducted by Jamovi (version 2.4.12).

## 3. Results

**Figure 2:**
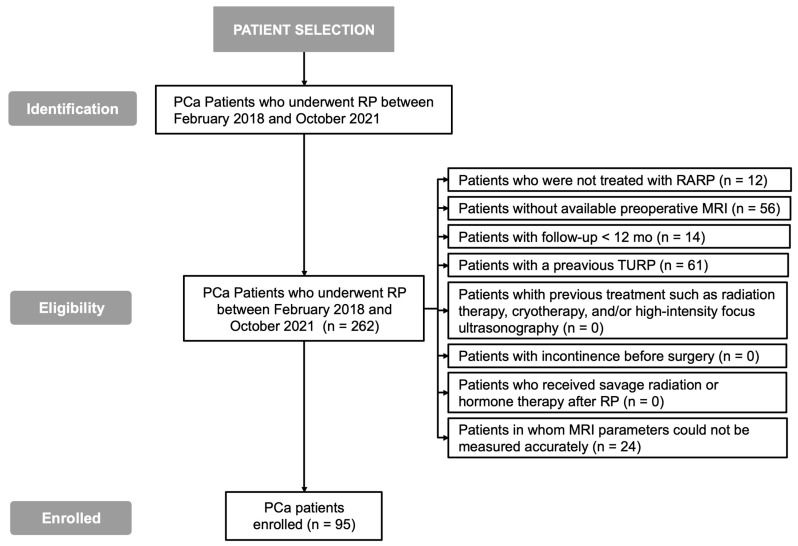
Patient selection flowchart.

A total of 262 PCa patients who underwent RP between February 2018 and October 2021 were identified (Figure 2). A total of 12 patients who did not undergo RARP, 56 patients without an available preoperative MRI, 14 patients with follow-up <12 months, 61 patients who underwent TURP, and 24 patients in whom MRI parameters could not be accurately measured were excluded. Therefore, 95 patients were enrolled.

The demographic and clinical characteristics of the study population are summarized in Table 1. The median age was 66 years (IQR 62–70), with a median height of 1.75 m (IQR 1.70–1.78), median weight of 80 kg (IQR 72–85), and a median BMI of 26.12 kg/m^2^ (IQR 23.88–28.09). The median PSA level was 7.5 ng/mL (IQR 5.5–9.6), while the median PV was 45 cc (IQR 34–59), resulting in a PSAD of 0.16 ng/mL/cc (IQR 0.10–0.26). Regarding baseline urinary symptoms, the median IPSS was 9 (IQR 6–14). QoL, assessed through the IPSS-related question, ranged from 0 in 23 patients (26.1%) to 6 in 2 patients (2.1%). At diagnosis, the majority of patients were classified as ISUP grade 2 (*n* = 59, 62.1%), followed by ISUP 3 (*n* = 29, 30.5%), ISUP 5 (*n* = 4, 4.2%), and ISUP 1 (*n* = 3, 3.2%). In terms of clinical staging, most patients were staged as cT2c (*n* = 63, 66.3%), while the remaining were distributed as follows: cT3c (*n* = 12, 12.6%), cT3a (*n* = 11, 11.6%), cT2a (*n* = 6, 6.3%), and cT2b (*n* = 3, 3.2%). Based on MRI findings, 26 patients (27.4%) were classified as LT A, 20 (21.1%) as LT B, 18 (18.9%) as LT C, and 31 (32.6%) as LT D. MRI also identified a median PUL of 36 mm (IQR 31–42), a median MUL of 15 mm (IQR 13–16), and a median IPP of 0 mm (IQR 0–0). Median ML was reported in 23 patients (24.2%). During follow-up, the proportion of patients regaining UC increased progressively over time. Immediately after VC removal, 32 patients (33.7%) had recovered UC. At 12 months postoperatively, UC recovery had occurred in 93 patients (97.8%).

Table 2, Table 3, Table 4, Table 5 and Table 6 show multivariate logistic regression results: only two models reported statistically significant results. LT C was a significant predictor of continence recovery at VC removal with respect to LT A in the model described in Table 2 (OR 0.1; 95% CI 0.04–0.71; *p*-value 0.01) (Table 2).

The value of IPSS was found to be a statistically significant predictor on continence at 1 month after RARP in the model in Table 6 (OR 1.08 (1.00–1.17; 0.04)).

No further statistical predictors were found for PUL, MUL, or ML presence, and IPP did not show any statistically significant impact on UC recovery in the models considered.

## 4. Discussion

This study aimed to evaluate the potential role of preoperative MRI-derived anatomic parameters in predicting UC recovery after RARP. Our findings suggest that only two variables were statistically associated with UC recovery at specific time points. First, at the time of VC removal, patients classified as having LT C on preoperative MRI showed a significantly higher likelihood of being continent compared to those with LT A. Second, an increase in preoperative IPSS was significantly associated with a reduced probability of UC recovery one month after RARP. However, no other variables, such as MUL, PUL, IPP, or the presence of ML, demonstrated a statistically significant correlation with UC recovery at any of the follow-up intervals considered.

These findings, although partially significant, may suggest that a clear and consistent association between UC recovery and preoperative MRI parameters is still elusive. One possible interpretation is that the anatomical characteristics evaluated may have a limited predictive role when considered in isolation, or that they primarily influence early UC recovery rather than long-term outcomes. Alternatively, the lack of broader statistical significance could reflect limitations in sample size, inter-observer variability in MRI interpretation, or the multifactorial nature of continence recovery after prostatectomy.

Our results are partially in line with previous studies in the literature, which show that urethral length, urethral sphincter dimensions, preoperative IPSS, and prostatic shape may be correlated to postoperative incontinence [28,29,30,31,32,33,34,35,36]. Sauer et al. evaluated the relationship between LT classification and UC recovery, reporting no significant differences in continence outcomes between different LT groups at 3 months (*p* = 0.24) and 6 months (*p* = 0.61) following RP [18]. Their findings support the hypothesis that LT may not have a durable or consistent impact on long-term continence outcomes. Similarly, Hikita et al. conducted a multivariate logistic regression analysis and found no statistically significant correlation between MUL and UC recovery, either at 1 month (OR 0.91; 95% CI 0.82–1.00; *p* = 0.06) or 12 months (OR 0.95; 95% CI 0.85–1.10; *p* = 0.39) postoperatively [23]. Interestingly, in their study, IPSS again emerged as a predictor of early continence: higher IPSS values were significantly associated with reduced UC recovery after one month (OR 1.14; 95% CI 1.02–1.28; *p* = 0.02), although not after 12 months (OR 0.99; 95% CI 0.87–1.14; *p* = 0.39) [4]. They also observed that patients with IPP greater than 5 mm experienced higher IPSS values postoperatively and had a lower rate of pad-free status at 1 month, suggesting that IPP might be used as a potential cutoff for identifying patients at high risk for early urinary incontinence.

On the other hand, some studies have yielded contrasting results, particularly regarding the role of MUL [17,24,26]. A recent meta-analysis highlighted a significant correlation between longer MUL and improved UC recovery at various time points [26]. Specifically, the analysis reported a hazard ratio (HR) of 1.05 (95% CI 1.02–1.08; *p* < 0.001) for early recovery, and higher odds ratios (ORs) for recovery at 1 month (OR 1.16; 95% CI 1.09–1.23; *p* < 0.001), 3 months (OR 1.08; 95% CI 1.03–1.14; *p* = 0.004), 6 months (OR 1.12; 95% CI 1.03–1.15; *p* < 0.001), and 12 months (OR 1.12; 95% CI 1.03–1.22; *p* = 0.006) following surgery. These findings suggest that MUL may serve as a robust and consistent predictor of both early and late UC recovery. Similar observations have been reported by other authors as well, reinforcing the importance of urethral length as a relevant anatomical factor [37]. Both the existing literature and our current findings indicate that patients undergoing nerve-sparing surgery achieve significantly better urinary continence outcomes compared to those without nerve preservation [38,39,40]. This advantage is likely related to the preservation of neurovascular structures that contribute to sphincter function and pelvic floor innervation, thereby facilitating earlier and more complete recovery of continence.

The present study must be interpreted in light of several limitations. Firstly, its retrospective design inherently introduces the risk of selection bias and limits the strength of causal inferences. Secondly, although all patients underwent preoperative MRI, image acquisition was not centralized, and inter-reader variability in LT classification or measurement of parameters such as MUL and IPP could have influenced the accuracy of data. Moreover, the relatively small sample size and the strict inclusion/exclusion criteria reduced the number of eligible patients, potentially impacting the statistical power of our analysis. It is also possible that the relatively low event rate in some follow-up intervals contributed to the lack of statistical significance in some variables.

Despite these limitations, our findings contribute to the ongoing discussion about the predictive role of anatomical features observed on preoperative MRI in determining postoperative continence outcomes. The heterogeneity of results reported in the literature underscores the complexity of UC recovery, which likely depends on a combination of anatomical, functional, surgical, and rehabilitative factors. Future prospective, multicentric studies with larger patient cohorts and standardized MRI protocols will be essential to clarify the true impact of specific MRI parameters—such as LT classification, MUL, and IPP—on continence recovery.

In this context, identifying patients at higher risk of persistent incontinence [31,41,42] is not only relevant for clinical prediction but also crucial for shared decision-making. Providing patients with accurate, personalized information about their individual risk of postoperative incontinence—based on their preoperative anatomical characteristics and functional status is essential to foster informed consent. A well-structured and transparent informed consent process [43,44,45,46] should include a discussion of these tailored risks, aligning surgical expectations and promoting early engagement in postoperative rehabilitation strategies when appropriate.

## 5. Conclusions

In conclusion, our study suggests that selected preoperative MRI-derived parameters, such as LT classification and IPSS, may influence early urinary continence recovery after RARP. In particular, patients who had Lee Type C might have higher probability of early urinary continence recovery. However, the lack of consistent associations with other anatomical variables highlights the need for larger, prospective multicenter studies to better define the predictive value of preoperative imaging in personalized patient management.

## Figures and Tables

**Table 1:** Patient characteristics.

Characteristics		Missing	Median (IQR); N (%)
**Age (yy)**		0	66 (62, 70)
**Weight (kg)**		0	80 (72, 85)
**Height (m)**		0	1.75 (1.70, 1.78)
**BMI (kg/m^2^)**		0	26.12 (23.88, 28.09)
**PSA (ng/mL)**		0	7.5 (5.5, 9.6)
**PV (cc)**		0	45 (34, 59)
**PSAD (ng/mL/cc)**		0	0.16 (0.10, 0.26)
**IPSS**		0	9 (6, 14)
**QoL**	0	7	23 (26.1%)
	1		19 (21.6%)
	2		16 (18.2%)
	3		9 (10.2%)
	4		11 (12.5%)
	5		8 (9.1%)
	6		2 (2.3%)
**Bladder neck sparing technique**		0	2 (2.1%)
**Nerve sparing technique**		0	79 (83.2%)
**ISUP**	1	0	3 (3.2%)
	2		59 (62.1%)
	3		29 (30.5%)
	4		0 (0.0%)
	5		4 (4.2%)
**Stage**	T2a	0	6 (6.3%)
	T2b		3 (3.2%)
	T2c		63 (66.3%)
	T3a		11 (11.6%)
	T3b		12 (12.6%)
**LT**	A	0	26 (27.4%)
	B		20 (21.1%)
	C		18 (18.9%)
	D		31 (32.6%)
**ML presence**		0	23 (24.2%)
**IPP (mm)**		0	0 (0, 0)
**PUL (mm)**		0	36 (31, 42)
**MUL (mm)**		0	15 (13, 16)
**Patients with UC**	baseline	0	32 (33.7%)
	1° month	0	75 (73.7%)
	3° month	0	81 (85.3%)
	6° month	0	86 (90.5%)
	12° month	0	93 (97.8%)

**Table 2:** Multivariate logistic regression analysis assessing the effect of independent variables (age, BMI, PV, IPSS, NS, ISUP, PUL) on urinary continence at the time of catheter removal and at 1, 3, and 6 months thereafter.

Variable		Odds Ratio (95% CI; *p*-Value)			
		VC Removal	1 Month	3 Months	6 Months
**Age (yy)**		0.96 (0.88–1.04; 0.28)	0.96 (0.89–1.05; 0.40)	1.00 (0.90–1.11; 0.99)	1.07 (0.93–1.24; 0.33)
**BMI (kg/m^2^)**		1.16 (0.99–1.35; 0.07)	1.16 (0.98–1.38; 0.09)	1.07 (0.87–1.31; 0.54)	1.24 (0.94–1.63; 0.13)
**PV (mL)**		1.01 (0.98–1.03; 0.54)	1.00 (0.97–1.03; 0.88)	1.00 (0.97–1.03; 0.85)	0.98 (0.93–1.03; 0.40)
**IPSS**		1.02 (0.95–1.09; 0.60)	1.08 (1.00–1.17; 0.05)	1.08 (0.98–1.18; 0.11)	1.09 (0.98–1.22; 0.12)
**Nerve sparing**	Yes	-	-	-	-
	No	0.50 (0.14–1.69; 0.26)	1.33 (0.36–4.95; 0.67)	2.15 (0.49–9.49; 0.31)	1.12 (0.16–8.00; 0.91)
**ISUP**	1	-	-	-	-
	2	1.04 (0.07–14.95; 0.98)	0.26 (0.01–4.96; 0.37)	0.16 (0.01–2.64; 0.20)	0.07 (0.00–1.86; 0.11)
	3	0.81 (0.05–13.48; 0.88)	0.47 (0.02–9.95; 0.63)	0.15 (0.01–3.13; 0.22)	0.06 (0.00–2.15; 0.12)
	5	1.80 (0.05–58.86; 0.74)	2.31 (0.07–80.75; 0.64)	2.15 (0.07–64.11; 0.66)	6.19 (0.14–283.38; 0.35)
**PUL (mm)**		0.96 (0.89–1.02; 0.20)	0.92 (0.85–1.00; 0.05)	0.97 (0.89–1.07; 0.56)	0.95 (0.84–1.08; 0.42)

**Table 3:** Multivariate logistic regression analysis assessing the effect of independent variables (age, BMI, PV, IPSS, NS, ISUP, MUL) on urinary continence at the time of catheter removal and at 1, 3, and 6 months thereafter.

Variable		Odds Ratio (95% CI; *p*-Value)			
		VC Removal	1 Month	3 Months	6 Months
**Age (yy)**		0.96 (0.87–1.05; 0.38)	0.97 (0.89–1.06; 0.49)	1.00 (0.90–1.11; 0.97)	1.08 (0.94–1.25; 0.26)
**BMI (kg/m^2^)**		1.16 (0.99–1.36; 0.07)	1.15 (0.97–1.36; 0.10)	1.07 (0.87–1.31; 0.54)	1.24 (0.94–1.63; 0.13)
**PV (mL)**		1.00 (0.98–1.02; 0.68)	0.99 (0.96–1.01; 0.28)	0.99 (0.96–1.02; 0.63)	0.96 (0.92–1.01; 0.16)
**IPSS**		1.01 (0.95–1.09; 0.67)	1.07 (0.99–1.16; 0.07)	1.07 (0.98–1.17; 0.12)	1.09 (0.98–1.22; 0.13)
**Nerve sparing**	Yes	-	-	-	-
	No	0.58 (0.17–1.95; 0.38)	1.59 (0.44–5.76; 0.48)	2.25 (0.51–9.98; 0.29)	1.33 (0.19–9.45; 0.77)
**ISUP**	1	-	-	-	-
	2	1.12 (0.08–15.22; 0.93)	0.41 (0.03–6.42; 0.52)	0.21 (0.01–3.32; 0.27)	0.07 (0.00–1.97; 0.12)
	3	0.94 (0.06–14.35; 0.97)	0.89 (0.05–15.23; 0.93)	0.21 (0.01–4.08; 0.30)	0.07 (0.00–2.32; 0.14)
	5	1.99 (0.06–64.02; 0.70)	2.95 (0.10–87.89; 0.53)	2.54 (0.09–74.27; 0.59)	6.89 (0.16–299.31; 0.32)
**MUL (mm)**		1.10 (0.94–1.29; 0.24)	1.05 (0.89–1.24; 0.60)	0.98 (0.80–1.20; 0.87)	1.10 (0.79–1.52; 0.57)

**Table 4:** Multivariate logistic regression analysis assessing the effect of independent variables (age, BMI, PV, IPSS, NS, ISUP, LT) on urinary continence at the time of catheter removal and at 1, 3, and 6 months thereafter.

Variable		Odds Ratio (95% CI; *p*-Value)			
		VC Removal	1 Month	3 Months	6 Months
**Age (yy)**		0.95 (0.88–1.04; 0.25)	0.97 (0.89–1.05; 0.42)	0.99 (0.89–1.10; 0.89)	1.08 (0.94–1.25; 0.28)
**BMI (kg/m^2^)**		1.16 (0.98–1.38; 0.09)	1.17 (0.98–1.39; 0.08)	1.05 (0.84–1.31; 0.65)	1.23 (0.93–1.63; 0.15)
**PV (mL)**		1.00 (0.98–1.02; 0.97)	0.99 (0.97–1.01; 0.31)	0.99 (0.97–1.02; 0.56)	0.97 (0.92–1.01; 0.17)
**IPSS**		1.02 (0.95–1.10; 0.56)	1.08 (1.00–1.16; 0.06)	1.07 (0.98–1.18; 0.15)	1.09 (0.97–1.24; 0.15)
**Nerve sparing**	Yes	-	-	-	-
	No	0.43 (0.12–1.58; 0.20)	1.53 (0.39–5.99; 0.54)	1.79 (0.36–8.89; 0.47)	1.07 (0.12–9.62; 0.95)
**ISUP**	1	-	-	-	-
	2	1.91 (0.13–28.21; 0.64)	0.45 (0.03–6.28; 0.55)	0.20 (0.01–3.16; 0.25)	0.12 (0.01–2.71; 0.18)
	3	1.67 (0.10–26.73; 0.72)	0.99 (0.07–14.79; 0.99)	0.21 (0.01–3.86; 0.29)	0.11 (0.00–3.33; 0.21)
	5	3.48 (0.10–124.57; 0.49)	3.20 (0.11–91.94; 0.50)	2.35 (0.07–75.97; 0.63)	11.86 (0.23–612.0.5; 0.22)
**LT**	A	-	-	-	-
	B	0.39 (0.10–1.55; 0.18)	2.06 (0.50–8.54; 0.32)	2.03 (0.40–10.22; 0.39)	0.77 (0.08–7.35; 0.82)
	C	0.17 (0.04–0.71; 0.01)	1.88 (0.41–8.68; 0.42)	1.48 (0.25–8.93; 0.67)	0.30 (0.02–4.33; 0.38)
	D	0.63 (0.16–2.50; 0.51)	1.57 (0.38–6.51; 0.54)	0.57 (0.08–4.10; 0.58)	0.78 (0.08–7.26; 0.83)

**Table 5:** Multivariate logistic regression analysis assessing the effect of independent variables (age, BMI, PV, IPSS, NS, ISUP, ML presence) on urinary continence at the time of catheter removal and at 1, 3, and 6 months thereafter.

Variable		Odds Ratio (95% CI; *p*-Value)			
		VC Removal	1 Month	3 Months	6 Months
**Age (yy)**		0.95 (0.88–1.03; 0.23)	0.96 (0.89–1.05; 0.40)	1.00 (0.90–1.11; 0.99)	1.08 (0.94–1.24; 0.30)
**BMI (kg/m^2^)**		1.14 (0.98–1.34; 0.10)	1.14 (0.96–1.35; 0.13)	1.05 (0.85–1.30; 0.63)	1.23 (0.94–1.62; 0.13)
**PV (mL)**		1.01 (0.98–1.03; 0.49)	1.00 (0.97–1.03; 0.99)	1.00 (0.97–1.03; 0.98)	0.97 (0.92–1.02; 0.21)
**IPSS**		1.02 (0.95–1.10; 0.59)	1.08 (1.00–1.17; 0.05)	1.08 (0.98–1.18; 0.11)	1.09 (0.98–1.22; 0.12)
**Nerve sparing**	Yes	-	-	-	-
	No	0.58 (0.17–2.01; 0.39)	1.64 (0.44–6.21; 0.46)	2.34 (0.53–10.29; 0.26)	1.25 (0.18–8.88; 0.82)
**ISUP**	1	-	-	-	-
	2	1.89 (0.14–24.77; 0.63)	0.59 (0.04–8.50; 0.70)	0.22 (0.02–3.25; 0.27)	0.11 (0.01–2.14; 0.14)
	3	1.93 (0.13–28.37; 0.63)	1.65 (0.10–26.13; 0.72)	0.26 (0.01–4.54; 0.35)	0.10 (0.00–3.02; 0.19)
	5	2.22 (0.07–70.15; 0.65)	2.86 (0.10–84.60; 0.54)	2.22 (0.08–62.96; 0.64)	8.16 (0.20–334.34; 0.27)
**ML presence**	Yes	-	-	-	-
	No	2.76 (0.82–9.37; 0.10)	4.81 (0.95–24.41; 0.06)	2.43 (0.34–17.43; 0.38)	1.12 (0.10–13.21; 0.93)

**Table 6:** Multivariate logistic regression analysis assessing the effect of independent variables (age, BMI, PV, IPSS, NS, ISUP, IPP) on urinary continence at the time of catheter removal and at 1, 3, and 6 months thereafter.

Variable		Odds Ratio (95% CI; *p*-Value)			
		VC Removal	1 Month	3 Months	6 Months
**Age (yy)**		0.95 (0.88–1.03; 0.23)	0.96 (0.89–1.05; 0.39)	1.00 (0.90–1.11; 0.98)	1.08 (0.94–1.24; 0.27)
**BMI (kg/m^2^)**		1.15 (0.98–1.35; 0.08)	1.13 (0.96–1.34; 0.15)	1.06 (0.86–1.30; 0.61)	1.25 (0.95–1.65; 0.12)
**PV (mL)**		1.00 (0.98–1.03; 0.87)	1.00 (0.97–1.03; 0.99)	1.00 (0.96–1.03; 0.90)	0.96 (0.92–1.01; 0.16)
**IPSS**		1.02 (0.95–1.10; 0.59)	1.08 (1.00–1.17; 0.04)	1.08 (0.98–1.18; 0.11)	1.09 (0.97–1.22; 0.13)
**Nerve sparing**	Yes	-	-	-	-
	No	0.53 (0.16–1.77; 0.30)	1.43 (0.39–5.24; 0.59)	2.20 (0.50–9.64; 0.30)	1.31 (0.18–9.33; 0.79)
**ISUP**	1	-	-	-	-
	2	1.61 (0.13–21.10; 0.72)	0.58 (0.04–8.26; 0.69)	0.21 (0.01–3.14; 0.26)	0.10 (0.00–2.00; 0.13)
	3	1.46 (0.10–20.84; 0.78)	1.37 (0.09–21.29; 0.82)	0.22 (0.01–3.93; 0.31)	0.08 (0.00–2.46; 0.15)
	5	2.34 (0.08–72.51; 0.63)	2.87 (0.10–83.42; 0.54)	2.30 (0.08–65.19; 0.62)	9.21 (0.21–401.75; 0.25)
**IPP (mm)**		0.98 (0.89–1.07; 0.64)	0.90 (0.78–1.04; 0.16)	0.96 (0.83–1.11; 0.58)	1.04 (0.88–1.24; 0.63)

## Data Availability

The data supporting the findings of this study are available from the corresponding author upon reasonable request. The data are currently part of the institutional research database and are not publicly available due to privacy and ethical restrictions.

## Abbreviations

The following abbreviations are used in this manuscript:

95% CI	95% Confidence Interval
BMI	Body Mass Index
CT	Computerized Tomography
GS	Gleason Score
IPP	Intravesical Prostatic Protrusion
IPSS	International Prostate Symptoms Score
IQR	Inter-Quartile Range
LT	Lee Type
ML	Median Lobe
MRI	Magnetic Resonance Imaging
MUL	Membranous Urethra Length
NST	Nerve Sparing Technique
OR	Odds Ratio
PCa	Prostate Cancer
PET	Positron Emission Tomography
PSA	Prostate Specific Antigen
PSAD	PSA Density
PSMA	Prostate Specific Membrane Antigen
PUL	Prostatic Urethra Length
PV	Prostate Volume
QoL	Quality of Life
RARP	Robot-Assisted Radical Prostatectomy
RP	Radical Prostatectomy
TURP	Trans-Urethral Resection of the Prostate
UC	Urinary Continence
UI	Urinary Incontinence
VC	Vesical Catheter

## References

[1] MacLennan S., Azevedo N., Duncan E., Dunsmore J., Fullwood L., Lumen N., Plass K., Ribal M.J., Roobol M.J., Nieboer D. (2023). Mapping European Association of Urology Guideline Practice Across Europe: An Audit of Androgen Deprivation Therapy Use Before Prostate Cancer Surgery in 6598 Cases in 187 Hospitals Across 31 European Countries. Eur. Urol..

[2] O’Toole C.C., Boakye N.F., Hannigan A., Jalali A. (2025). Clinical impact of MRI-based risk calculators for prostate cancer diagnosis: A systematic review and meta-analysis. Prostate Cancer Prostatic Dis..

[3] Brant A., Campi R., Carrion D.M., Esperto F., Sze C., Johnson J.P., Hu J.C., Borregales L.D., ICE-OUT (International Collaboration on Experiences and Opinions of Urology Trainees) Collaborators (2022). Findings from an international survey of urology trainee experience with prostate biopsy. BJU Int..

[4] Mantica G., Pacchetti A., Aimar R., Cerasuolo M., Dotta F., Olivero A., Pini G., Passaretti G., Maffezzini M., Terrone C. (2018). Developing a five-step training model for transperineal prostate biopsies in a naïve residents’ group: A prospective observational randomised study of two different techniques. World J. Urol..

[5] Bauckneht M., Rebuzzi S.E., Ponzano M., Borea R., Signori A., Frantellizzi V., Rizzini E.L., Mascia M., Lavelli V., Miceli A. (2022). Prognostic Value of the BIO-Ra Score in Metastatic Castration-Resistant Prostate Cancer Patients Treated with Radium-223 after the European Medicines Agency Restricted Use: Secondary Investigations of the Multicentric BIO-Ra Study. Cancers.

[6] Liu B., Cai Q., Zhao X., Su H., Lin Z., Wu J., Li X., Zhu W., Zou C., Luo Y. (2025). A nomogram based on radiomic features from peri-prostatic adipose tissue for predicting bone metastasis in first-time diagnosed prostate cancer patients. Adipocyte.

[7] De Nunzio C., Lombardo R., Baldassarri V., Cindolo L., Bertolo R., Minervini A., Sessa F., Muto G., Bove P., Vittori M. (2021). Rotterdam mobile phone app including MRI data for the prediction of prostate cancer: A multicenter external validation. Eur. J. Surg. Oncol. (EJSO).

[8] Mantica G., Malinaric R., Dotta F., Paraboschi I., Guano G., Rebuffo S., Garriboli M., Suardi N., Van der Merwe A., Terrone C. (2020). Urology apps: Overview of current types and use. Cent. Eur. J. Urol..

[9] Telecan T., Chiorean A., Sipos-Lascu R., Caraiani C., Boca B., Hendea R.M., Buliga T., Andras I., Crisan N., Lupsor-Platon M. (2025). ISUP Grade Prediction of Prostate Nodules on T2WI Acquisitions Using Clinical Features, Textural Parameters and Machine Learning-Based Algorithms. Cancers.

[10] Fredman E., Tschernichovsky R., Shemesh D., Weinstock-Sabbah M., Azuz R.D., Radus R., Moore A., Limon D. (2025). Stereotactic Radiotherapy to the Prostate and Pelvic Lymph Nodes for High-Risk and Very High-Risk Prostate Cancer in a Setting with a Hydrogel Spacer: A Toxicity Report. Cancers.

[11] Kwon W.-A., Joung J.Y. (2025). T-Cell Engager Therapy in Prostate Cancer: Molecular Insights into a New Frontier in Immunotherapy. Cancers.

[12] Sharifi M., Armstrong C.M., Ning S., Leslie A.R., Schaaf Z.A., Maine J.P., Lou W., Li P.-K., Xu H., Liu C. (2025). Steroid Sulfatase Regulates Metabolic Reprogramming in Advanced Prostate Cancer. Cancers.

[13] De Marchi D., Mantica G., Tafuri A., Giusti G., Gaboardi F. (2022). Robotic surgery in urology: A narrative review from the beginning to the single-site. AME Med. J..

[14] Valenzi F.M., Santarelli V., Avesani G., Aljoulani M., Haberal H.B., Anguiano J.R.T., Morgantini L.A., Calvo R.S., Biasatti A., Fuschi A. (2025). Single-Port Versus Multi-Port Robotic Radical Prostatectomy in Elderly Patients. Cancers.

[15] Robert G., Blin P., Bladou F., Jové J., Ouattara E., Rouyer M., Droz-Perroteau C., Piazza L., Preaubert N. (2025). Comparative effectiveness of robot-assisted vs. open prostatectomy: A real-life nationwide study. World J. Urol..

[16] Marchioni M., Primiceri G., Castellan P., Schips L., Mantica G., Chapple C., Papalia R., Porpiglia F., Scarpa R.M., Esperto F. (2020). Conservative management of urinary incontinence following robot-assisted radical prostatectomy. Minerva Urol. Nephrol..

[17] Tienza A., Hevia M., Benito A., Pascual J.I., Zudaire J.J., Robles J.E. (2015). MRI factors to predict urinary incontinence after retropubic/laparoscopic radical prostatectomy. Int. Urol. Nephrol..

[18] Sauer M., Tennstedt P., Berliner C., Well L., Huland H., Budäus L., Adam G., Beyersdorff D. (2019). Predictors of short and long term urinary incontinence after radical prostatectomy in prostate MRI: Significance and reliability of standardized measurements. Eur. J. Radiol..

[19] Castellan P., Ferretti S., Litterio G., Marchioni M., Schips L. (2023). Management of Urinary Incontinence Following Radical Prostatectomy: Challenges and Solutions. Ther. Clin. Risk Manag..

[20] Santos N.A.D.S.E., Saintrain M.V.D.L., Regadas R.P., Da Silveira R.A., De Menezes F.J.C. (2017). Assessment of Physical Therapy Strategies for Recovery of Urinary Continence after Prostatectomy. Asian Pac. J. Cancer Prev..

[21] Szczygielska D., Knapik A., Pop T., Rottermund J., Saulicz E. (2022). The Effectiveness of Pelvic Floor Muscle Training in Men after Radical Prostatectomy Measured with the Insert Test. Int. J. Environ. Res. Public Heal..

[22] Gurwin A., Kowalczyk K., Knecht-Gurwin K., Stelmach P., Nowak Ł., Krajewski W., Szydełko T., Małkiewicz B. (2022). Alternatives for MRI in Prostate Cancer Diagnostics—Review of Current Ultrasound-Based Techniques. Cancers.

[23] Hikita K., Honda M., Teraoka S., Nishikawa R., Kimura Y., Tsounapi P., Iwamoto H., Morizane S., Takenaka A. (2020). Intravesical prostatic protrusion may affect early postoperative continence undergoing robot-assisted radical prostatectomy. BMC Urol..

[24] Mac Curtain B.M., Sugrue D.D., Qian W., O’CAllaghan M., Davis N.F. (2023). Membranous urethral length and urinary incontinence following robot-assisted radical prostatectomy: A systematic review and meta-analysis. BJU Int..

[25] Matsushita K., Kent M.T., Vickers A.J., von Bodman C., Bernstein M., Touijer K.A., Coleman J.A., Laudone V.T., Scardino P.T., Eastham J.A. (2015). Preoperative predictive model of recovery of urinary continence after radical prostatectomy. BJU Int..

[26] Mungovan S.F., Sandhu J.S., Akin O., Smart N.A., Graham P.L., Patel M.I. (2017). Preoperative Membranous Urethral Length Measurement and Continence Recovery Following Radical Prostatectomy: A Systematic Review and Meta-analysis. Eur. Urol..

[27] von Bodman C., Matsushita K., Savage C., Matikainen M.P., Eastham J.A., Scardino P.T., Rabbani F., Akin O., Sandhu J.S. (2012). Recovery of Urinary Function After Radical Prostatectomy: Predictors of Urinary Function on Preoperative Prostate Magnetic Resonance Imaging. J. Urol..

[28] Jacobsen N.-E.B., Moore K.N., Estey E., Voaklander D. (2007). Open Versus Laparoscopic Radical Prostatectomy: A Prospective Comparison of Postoperative Urinary Incontinence Rates. J. Urol..

[29] Gordon A., Skarecky D.W., Ahlering T. (2014). Long-term Outcomes in Severe Lower Urinary Tract Symptoms in Men Undergoing Robotic-assisted Radical Prostatectomy. Urology.

[30] Gacci M., De Nunzio C., Sakalis V., Rieken M., Cornu J.-N., Gravas S. (2023). Latest Evidence on Post-Prostatectomy Urinary Incontinence. J. Clin. Med..

[31] Coakley F.V., Eberhardt S., Kattan M.W., Wei D.C., Scardino P.T., Hricak H. (2002). Urinary continence after radical retropubic prosta-tectomy: Relationship with membranous urethral length on preoperative endorectal magnetic resonance imaging. J Urol..

[32] Lee S.E., Byun S.-S., Lee H.J., Song S.H., Chang I.H., Kim Y.J., Gill M.C., Hong S.K. (2006). Impact of variations in prostatic apex shape on early recovery of urinary continence after radical retropubic prostatectomy. Urology.

[33] Hoeh B., Wenzel M., Müller M., Wittler C., Schlenke E., Hohenhorst J.L., Köllermann J., Steuber T., Graefen M., Tilki D. (2022). Urethral Sphincter Length but Not Prostatic Apex Shape in Preoperative MRI Is Associated with Mid-Term Continence Rates after Radical Prostatectomy. Diagnostics.

[34] Boellaard T.N., Haan M.C.v.D.-D., Heijmink S.W.T.P.J., Tillier C.N., Veerman H., Mertens L.S., van der Poel H.G., van Leeuwen P.J., Schoots I.G. (2023). Membranous urethral length measurement on preoperative MRI to predict incontinence after radical prostatectomy: A literature review towards a proposal for measurement standardization. Eur. Radiol..

[35] Haan M.C.v.D.-D., Boellaard T.N., Tissier R., Heijmink S.W., van Leeuwen P.J., van der Poel H.G., Schoots I.G. (2022). Value of Different Magnetic Resonance Imaging-based Measurements of Anatomical Structures on Preoperative Prostate Imaging in Predicting Urinary Continence After Radical Prostatectomy in Men with Prostate Cancer: A Systematic Review and Meta-analysis. Eur. Urol. Focus..

[36] Iacovelli V., Carilli M., Sandri M., Forte V., Cipriani C., Bertolo R., Vittori M., Petta F., Maiorino F., Signoretti M. (2022). The role of preoperative prostatic shape in the recovery of urinary continence after robotic radical prostatectomy: A single cohort analysis. Prostate Cancer Prostatic Dis..

[37] Lamberg H., Shankar P.R., Singh K., Caoili E.M., George A.K., Hackett C., Johnson A., Davenport M.S. (2022). Preoperative Prostate MRI Predictors of Urinary Continence Following Radical Prostatectomy. Radiology.

[38] Maruo M., Goto Y., Miyazaki K., Inoue A., Kurokawa K., Enomoto A., Tanaka S., Katsura S., Sugawara S., Fuse M. (2024). Novel nerve-sparing robot-assisted radical prostatectomy with endopelvic fascia preservation and long-term outcomes for a single surgeon. Sci. Rep..

[39] Xiang P., Du Z., Guan D., Yan W., Wang M., Guo D., Liu D., Liu Y., Ping H. (2024). Is there any difference in urinary continence between bilateral and unilateral nerve sparing during radical prostatectomy? A systematic review and meta-analysis. World J. Surg. Oncol..

[40] Reeves F., Preece P., Kapoor J., Everaerts W., Murphy D.G., Corcoran N.M., Costello A.J. (2015). Preservation of the Neurovascular Bundles Is Associated with Improved Time to Continence After Radical Prostatectomy But Not Long-term Continence Rates: Results of a Systematic Review and Meta-analysis. Eur. Urol..

[41] Yamashita K., Kijima Y., Sekido E., Nagasaka N., Inui M. (2023). Predictors of Long-Term Urinary Incontinence After Robot–Assisted Laparoscopic Prostatectomy. Res. Rep. Urol..

[42] Kachlik D., Baca V., Stingl J., Sosna B., Lametschwandtner A., Minnich B., Setina M. (2007). Architectonic Arrangement of the Vasa Vasorum of the Human Great Saphenous Vein. J. Vasc. Res..

[43] Irem D., Salmi A.R., Jamina N., Ait Houssa M., Bensghir M., Haimeur C., Lalaoui S.J., Azendour H., Kamili N.D. (2015). Quality of an Informed Consent Prior to a Surgical Intervention. Patient Saf. Qual. Improv..

[44] Agozzino E., Borrelli S., Cancellieri M., Carfora F.M., Di Lorenzo T., Attena F. (2018). Does written informed consent adequately inform surgical patients?. BMC Med. Ethics.

[45] Raper S.E.M., Clapp J.T., Fleisher L.A. (2021). Improving Surgical Informed Consent. Ann. Surg. Open.

[46] Convie L.J., Carson E., McCusker D., McCain R.S., McKinley N., Campbell W.J., Kirk S.J., Clarke M. (2020). The patient and clinician experience of informed consent for surgery. BMC Med. Ethics..

